# Protocol for the Cognitive Interventions and Nutritional Supplements (CINS) trial: A randomized controlled multicenter trial of a brief intervention (BI) versus a BI plus cognitive behavioral treatment (CBT) versus nutritional supplements for patients with long-lasting muscle and back pain

**DOI:** 10.1186/1471-2474-12-152

**Published:** 2011-07-07

**Authors:** Silje E Reme, Torill H Tveito, Trudie Chalder, Tormod Bjørkkjær, Aage Indahl, Jens I Brox, Egil Fors, Eli M Hagen, Hege R Eriksen

**Affiliations:** 1 Uni Health, Uni Research, Bergen, Norway; 2 Harvard School of Public Health, Boston, MA, USA; 3 Department of Health Promotion and Development, Faculty of Psychology, University of Bergen, Norway; 4 Department of Psychological Medicine, King's College London, UK; 5 National Institute of Nutrition and Seafood Research (NIFES), Bergen, Norway; 6 Department of Psychiatry, Haukeland University Hospital, Bergen, Norway; 7 Department of Orthopedics, Oslo University Hospital, Oslo, Norway; 8 Department of Physical Medicine and Rehabilitation, Sykehuset Innlandet HF, Ottestad, Norway; 9 Department of Psychiatry, St Olav University Hospital, Trondheim, Norway; 10 Department of Public Health and General Practice, Faculty of Medicine, Norwegian University of Science and Technology, Trondheim, Norway; 11 Center for Pain and Complex Disorders, Department of Anaesthesia, St Olav University Hospital, Trondheim, Norway; 12 Clinic of Physical Medicine and Rehabilitation, Vestfold Hospital Trust, Stavern, Norway

## Abstract

**Abstract:**

**Background**

Brief intervention programs are clinically beneficial, and cost efficient treatments for low back pain, when offered at 8-12 weeks, compared with treatment as usual. However, about 30% of the patients do not return to work. The European Guidelines for treatment of chronic low back pain recommends Cognitive Behavioral Therapy (CBT), but conclude that further research is needed to evaluate the effectiveness of CBT for chronic low back pain.

**Methods/Design**

The aim of the multicenter CINS trial (Cognitive Interventions and Nutritional Supplements) is to compare the effectiveness of 4 different interventions; Brief Intervention, Brief Intervention and CBT, Brief Intervention and nutritional supplements of seal oil, and Brief Intervention and nutritional supplements of soy oil. All participants will be randomly assigned to the interventions. The nutritional supplements will be tested in a double blind design. 400 patients will be recruited from a population of chronic low back pain patients that have been sick listed for 2-10 months. Four outpatient clinics, located in different parts of Norway, will participate in recruitment and treatment of the patients.

The Brief Intervention is a one session cognitive, clinical examination program based on a non-injury model, where return to normal activity and work is the main goal, and is followed by two booster sessions. The CBT is a tailored treatment involving 7 sessions, following a detailed manual. The nutritional supplements consist of a dosage of 10 grams of either soy or seal oil (capsules) per day for 3 months, administered in a double blind design. All patients will be followed up with questionnaires after 3, 6 and 12 months, while sick leave data will be collected up to at least 24 months after randomization. The primary outcome of the study is sick leave and will be based on register data from the National Insurance Administration. Secondary outcomes include self-reported data on disability, pain, and psychological variables.

**Conclusions**

To our knowledge, the CINS trial will be the largest, randomized trial of psychological and nutritional interventions for chronic low back pain patients to date. It will provide important information regarding the effectiveness of CBT and seal oil for chronic low back pain patients.

**Trial Registration:**

http://www.clinicaltrials.gov, with registration number NCT00463970.

## Background

Muscle pain including low back pain (LBP) is common in the general population, and accounts for about 50% of the long-term sick leave in Norway. It is also among the most common reasons for frequent visits to the general practitioner [1]. Lack of knowledge related to the understanding of pain mechanisms, treatment strategies and management of back pain contribute to the problem [2]. The transition from normal complaints to prolonged illness and disability is poorly understood, but there are new studies suggesting augmented central pain processing [3]. This transition has recently been attributed to neurobiological and psychobiological sensitization mechanisms [4]. The theoretical and empirical background for this hypothesis has been developed in some detail. The main foundation is a general and systematic stress theory, the Cognitive Activation Theory of Stress, (CATS), developed from empirical data from human and animal studies [5].

The treatment principles for LBP and for other types of non-specific muscle pain, have changed over the last 15 to 20 years, from traditional treatments like bed rest and inactivity to more active treatment strategies ("The back pain revolution")[6]. Brief intervention programs have been demonstrated to be clinically beneficial and cost effective [7]. The prognosis for patients with chronic low back pain (CLBP) is poor when the condition has lasted over an extended period of time [8,9].

### Non-specific pain with high levels of comorbidity

For most patients with LBP, a precise pathoanatomical diagnosis is often impossible due to weak associations between symptoms and anatomical findings [10]. A high degree of comorbidity has been described in patients with non-specific LBP [11]. In a survey of 457 patients referred to a spine clinic, less than 2% of the patient population reported LBP as their only complaint [12]. A substantial proportion of the patients reported a significant number of other subjective health complaints, including widespread muscle pain as well [12]. This is consistent with previous Danish and Norwegian data [13,14] and recent findings in the US [11]. In these studies, high levels of comorbidity were associated with a poor prognosis. Comorbidity seems to be a general phenomenon in patient groups with unspecific conditions and subjective health complaints, or "medically unexplained symptoms" such as irritable bowel syndrome [15]. The high number of different subjective health complaints in these conditions and reports of the effectiveness of psychological treatments, such as CBT in several of these patient groups [16-18], suggest that there are common psychobiological elements and less specificity than the diagnostic labels suggest [3].

### Theories of chronic pain

It is well recognized that the relationship between pathology and impairment are influenced by psychosocial variables. CLBP is therefore often viewed as a biopsychosocial phenomenon, where biological, psychological and social factors interact and mutually influence each other [19]. Avoidance may be an adaptive response to acute pain. However, sustained avoidance of movement, social interactions, leisure activities, and work, may increase pain and disability [20]. A number of models have evolved over the years to explain chronic pain behavior. These include the Gate Control Theory of Pain [21], operant conditioning paradigms [22], fear avoidance models [20,23,24], and acceptance models [25,26]. All of these models are brought together within the Cognitive Activation Theory of Stress (CATS) [5], which assumes that acquired response and stimulus expectancies determine the physiological and psychological responses, which in turn influence subjective health complaints such as pain through neurobiological sensitization [4,5].

### Evidence based treatments for LBP

The treatment principles applied in this study follow the evidence-based guidelines for the treatment of CLBP, developed in Europe (http://www.backpaineurope.org)[27]. The recommended treatments include conservative treatments like CBT, supervised exercise therapy, brief educational interventions (BI), and multidisciplinary bio-psychosocial treatments. A recent review of psychological treatments for CLBP, provide support for the efficacy of psychological interventions, including CBT, among persons with CLBP [28]. While the evidence points to BI and CBT as effective treatments, many of the studies included in the review, are at risk of bias because of methodological problems, and it has been argued that confidence in the results are unwarranted [29]. BI's are associated with positive outcomes for return to work early in the process [30]. However, it is still unclear whether the effect would be as beneficial later on, when the patients may develop behavior that adversely affects their illness and recovery. Some studies have shown that CBT is effective in reducing pain, while other studies have shown beneficial effects on physical and mental function and sick leave rates, but not pain [31,32]. It is difficult to replicate studies and results of the effects of CBT, because the components of the therapy and the experience of the therapists vary, and the treatment protocols are rarely documented. The European Guidelines for treatment of chronic low back pain emphasize the need to evaluate the effectiveness of using CBT-therapists without formal training in clinical psychology. These issues will be addressed and answered in the current trial; the CBT treatment protocol will be documented in a manual, and the treatment will be monitored closely. Therapists with different professional backgrounds will be recruited, trained, and followed in order to apply the protocol.

### Nutritional supplements

Seal oil is a marine oil that is relatively rich in the omega-3 polyunsaturated fatty acids eicosapentaenoic acid (EPA; 20:5n-3), docosapentaenoic acid (DPA; 22:5n-3) and docosahexaenoic acid (DHA, 22:6n-3). It is promoted commercially for its allegedly, beneficial effects on joint and muscle pain. There is little scientific documentation of the effect of seal oil, apart from an indication of a positive effect on muscle pain in a few Norwegian studies [33-36] of patients with inflammatory bowel disease and psoriatic arthritis. There are no clinical trials of the effectiveness of seal oil on patients with low back pain or unspecific musculoskeletal complaints as their main problem.

Pain and the immune system have many signal substances in common. Potentially algogenic substances are released from immune cells into inflamed tissues, i.e. cytokines from mast cells or TNFα from macrophages. Antagonism or neutralization of TNFα reduces pain and hyperalgesia in many animal models of inflammation. TNFα antibodies or neutralizing agents have been a successful therapy for pain relief in many autoimmune disorders such as arthritis, psoriasis, ankylosing spondylitis, and Crohn's disease [37]. Dietary fatty acids are incorporated into cell membranes of blood and tissues, where arachidonic acid (AA; 20:4n-6) generally prevails as a result of the Western diet where its precursor linoleic acid (18:2n-6) is in grand supply [38]. AA and the n-3 PUFA EPA, DPA and DHA, found mainly in fatty fish and marine oils, are substrates for various pro- and anti-inflammatory lipid mediators respectively [38]. Eicosanoids are a group of hormone-like compounds involved in both pro-inflammatory and anti-inflammatory processes and suppression of the immune system. As a general rule, eicosanoids derived from arachidonic acid (AA) are more potent triggers of immunological responses than eicosanoids derived from eicosapentaenoic acid (EPA, 20:5n-3) [38], though recently AA has been shown to have anti-inflammatory effects as well as the above n-3 PUFAs [38,39]. Soy oil is a vegetable oil rich in LA. It also contains some α-linolenic acid (18:3n-3), which is the precursor of EPA, but direct intake of marine oils, fish or other seafood is generally necessary because the conversion of α-linolenic acid to EPA and DHAis limited [40]. Lowering the ratio of n-6 to n-3 PUFAs to suppress n-6 eicosanoids and proinflammatory cytokines, may be important for pain relief in chronic inflammatory disorders since western diet is dominated by linoleic acid and AA [41].

### Cortisol

Cortisol is accepted as a robust marker of activation of adrenocortical activity (HPA activity) and follows a 24 hour rhythm with low levels in the evening and a characteristic peak level, usually found the first hour after awakening [42,43]. Short-term activation of the system is necessary and adaptive. Sustained activation of the system occurs when the individual does not expect to cope with a situation, and may be associated with negative health outcomes [5]. A flat cortisol curve, with a low deviation from morning to evening and high evening values may be an indication of insufficient recovery or sustained activation [44]. High levels of cortisol in the evening have been found in patients with chronic widespread pain [45] and chronic fatigue syndrome [46]. In one recent study, maladaptive coping strategies were negatively correlated to the cortisol response after awakening in CLBP patients [47].

### Predictors of outcome

A number of different factors contribute to the treatment success of interventions for LBP. The most important factors seems to be subjective ratings of pain intensity and disability, affective parameters, pain related cognitions, health control beliefs, and coping strategies [2,48-51]. More objective parameters, like medical data and objective work-related factors, appear less important in predicting treatment outcome [48,52]. It appears that subjective evaluations of health status and job satisfaction are more important predictors of the patients' likelihood of returning to work after sick leave, than the physical aspects of disability and job demands [53]. One of the strongest predictors of return to work seems to be the patients' own belief in return to work [51,54-57].

## Methods/Design

### Aims

**Figure 1:**
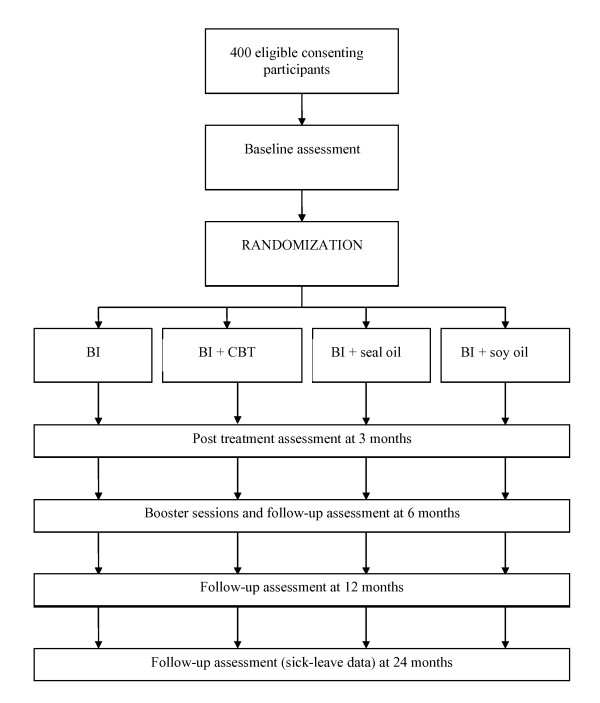
**Flowchart of trial design**.

The main goal in this study is to test out if a systematic CBT or nutritional supplements have additional effects on Brief Intervention (BI) in patients sick listed between 2 and 10 months for unspecific LBP. The patients will be randomized to BI only, BI and CBT, BI and seal oil, or BI and soy oil (Figure 1).

### Objectives

The CINS trial is designed to answer the following questions:

#### Primary objectives

1) Are BI and CBT, and/or BI and seal oil, more effective than BI alone (BI and placebo) in increasing return to work, reducing health complaints, or both?

2) Are BI and CBT more effective than BI and seal oil, in increasing return to work, reducing health complaints, or both?

#### Secondary objectives

The secondary analyses are exploratory, but will be guided by previous findings.

1) What are the relative cost-effectiveness and cost-benefit of these interventions?

2) What is the prevalence of psychiatric disorders in this population of chronic LBP patients?

3) What baseline factors predict different treatment outcomes in the different intervention groups?

4) Will any of the interventions impact the cortisol profiles of the LBP patients?

### Hypotheses of efficacy

1) BI and CBT will be superior in return to work and reduction of sick leave

2) BI and seal oil will reduce health complaints, but will not influence return to work or sick leave

### Number and source of participants

The goal is to recruit 400 participants (age between 20 and 60 years) to the trial (see sample size calculations). The Norwegian National Insurance Administration (NAV) will send a letter of invitation to patients that have been at least 50% sick listed, for 2 to 10 months due to LBP. In Norway, at any point in time, there are approximately 14000 patients on sick leave for 6 months, due to low back pain (Brage, NAV, personal communication). Patients on sick leave that geographically belong to one of the participating clinics are invited to participate in the study. The letters of invitation will be sent from NAV, and will include information about the study, and a response sheet. If a patient is interested in participating in the study, he or she fills out their contact information on the response sheet, and returns it to the research unit (Uni Health). After the response sheet has been received, the patients will be invited to the participating local back pain/pain clinic for a final evaluation for inclusion into the study. For those included, baseline assessment, BI, and randomization to any of the four interventions will be performed.

### Duration

Treatment according to randomization starts as soon as possible after allocation, with a maximum of 2 weeks delay. The main outcome assessment will be one year after randomization, but data on sick leave and other social benefits will be followed for at least another year.

### Inclusion criteria

1) Sick leave due to LBP for 2-10 months

2) At least 50% sickness compensation

3) Both participant and clinician agree that randomization is acceptable

4) Written informed consent from the participant

5) At least 50% employed

6) One of the following ICPC diagnoses: L02, L03, L84, or L86

7) Age between 20 and 60 years

### Exclusion criteria

1) Less than 50% sick listed or not on sick leave anymore

2) Pregnancy

3) Hemophilia

4) Osteoporosis (known osteoporotic fracture, or on anti osteoporostic medication)

5) Currently being treated for cancer

6) Recent back trauma

7) Serious psychiatric disorders (mainly due to ongoing psychosis, high suicide risk, and/or serious depression), assumed to be incompatible with participation in the trial.

8) Not fluent in Norwegian (assumed to be incompatible with CBT)

9) Debilitating Cardiovascular disease

10) Patients on warfarin treatment (blood thinner, e.g. Marevan)

11) Ongoing insurance trial, lawsuit, or pending legal action for LBP or related conditions

### Baseline assessment

At the clinic, the patients will be informed of their rights according to the Helsinki declaration, receive additional information about the study, and sign a written informed consent form before any trial related procedure takes place. The design of the trial will be explained to the patients in detail; e.g. with emphasis on the potential advantages of randomization, a description of each active intervention, consequences of participation, potential side effects of any of the treatments, and the necessity of the blinded nutritional supplement procedure. Then the patients will fill out the questionnaires, and a blood sample will be drawn to determine the baseline level of fatty acid profile in a selection of the participants. All patients will receive the Brief Intervention (BI), consisting of a clinical examination by a medical doctor (MD), and follow-up by a physiotherapist. During the clinical examination the MD will screen the patients according to the selection criteria. The MDs will also screen for psychiatric pathology, and exclude patients with serious psychopathology assumed to be incompatible with participation in the trial. The Mini-International Neuropsychiatric Interview (M.I.N.I) [58] will be used for this screening. After the patients have received the BI treatment, all eligible patients will be randomized to one of four treatment groups.

### Randomization

The patients will be randomized, using concealed randomization, to one of the following four treatments; 1) BI only, 2) BI and CBT, 3) BI and seal oil, or 4) BI and soy oil. This is done according to a computer generated randomization list, generated by the trial statistician. The list is stratified by clinic and gender. A central telephone randomization system, as already set up by our research unit will be used. At each of the participating clinics, a research assistant that is not involved in the treatment calls the research unit (Uni Health) to get information on which treatment the patient is randomized to. The research unit receives information about the patient's id number, gender, age, and diagnoses before the treatment allocation is disclosed, ensuring that the sequence is concealed until the intervention is assigned. The allocation code, including details of block size etc. will not be revealed to the researchers or the clinicians until recruitment, data collection, and laboratory analyses are complete. For those patients allocated to nutritional supplementation, the clinics provide blinded boxes, containing capsules with either seal oil or soy oil. All researchers, clinicians, and participants are blinded to treatment allocation of individual participants for the nutritional supplementation. Researchers at Uni Health, the trial statistician, and those assessing the main outcome of the study will be blinded to group assignment. Patients and CBT clinicians will not be blinded to group assignment.

### Ethical considerations

The Norwegian Regional Ethical Committee and the Norwegian Social Science Data Services have approved the study. The research will be carried out in compliance with the Helsinki declaration. Personal confidentiality is guaranteed, and declarations of voluntary participation with detailed information on the study processes and purposes, will be signed by each participant, emphasizing the right to withdraw from the experiment at any time without any explanation. We find the randomization procedure ethically acceptable because BI has been shown to be effective, and all patients will receive that treatment. However, the effect of BI together with a prolonged and systematic CBT for this patient group has not been studied. This study has a more systematic and consistent theoretical foundation, including a systematic manual for the therapy, than previous studies. Previous studies on BI have been performed in patients at an earlier stage of LBP. The treatment with seal oil and soy oil is beneficial or inert, but there are no RCTs on the possible effect in patients with LBP. The evidence of effect of seal oil is sufficiently demonstrated to do the experiment, and the oil is already in use in other controlled pilot trials.

### Treatment interventions

#### 1. BI (Brief intervention)

BI is a one session cognitive, clinical examination program based on a non-injury model, where return to normal activity and work is the main goal. Previous studies of BI for LBP have shown significant reduction of sick leave compared to treatment as usual [7,30,59-61]. A treatment manual for the BI is written. Consensus between all participating BI-clinicians was reached about the final version of the treatment manual. The essential feature of the intervention is the use of an eclectic cognitive and educational approach, throughout a thorough medical examination, conducted by a specialist in physical medicine and rehabilitation. The intervention includes diagnostic clarification, reassurance about normal findings, and encouragement to engage in physical activity as normal as possible. BI was checked against the Cognitive Therapy Adherence and Competence Scale (CTACS) [62], and according to CTACS it can not be categorized as a full-scale CBT. Patients will be guided towards a new understanding of the back pain, and given practical advice about how their back function may be improved. The examination is thorough, with detailed feedback on any medical findings and normal functions, and clear and consistent explanations on pain and defense mechanisms. If any "red flags" are identified, indicating serious pathology, the patients will be referred appropriately and excluded from the study. Neurological examinations will be conducted, and information regarding the importance of the findings will be given (e.g. positive comments about normal findings such as normal nerve function in legs). Painful muscles will be identified. If the patient moves in a tense way, attention is given to make the patient aware of how muscles may become dysfunctional, and how this may be maintained, and possibly worsen the condition. If the patient brings medical images, these are demonstrated and explained. Patients are told that looking for the source of pain on radiographs have limited importance; degenerative changes in the spine are most often normal aging processes and not necessarily painful. They are informed about the good prognosis, and the importance of staying active to avoid development of muscle dysfunction. They are further reassured that light activity will not be harmful to their backs, but on the contrary, light activity is more likely to improve their condition. The main purpose of the intervention is to provide the patients with coping skills to manage their back pain through evidence based information, practical advice and reassurance, and to motivate and encourage them to stay active, despite the pain. All personnel involved with the patients will give them the same consistent message. After the medical examination, the patients will receive a follow-up session with a physiotherapist, involving an educational and a behavioral part. The purpose of the *education *is to strengthen the message given in the medical examination. The purpose of the *behavioral *component is to help the patient turn the new insight into practical action. Patients will be encouraged to contact the spine clinic whenever they want. They will also receive two booster sessions to ensure that they still have the insight and understanding of their condition, and that they are able to cope with it. The first booster is given within 2 weeks, while the other is given 6 months after the first BI session. The booster sessions will last about 10-15 minutes. The results from the examination will be sent to the patients' primary care physician.

#### 2. BI and CBT

Seven sessions of individual CBT, over a period of 2-3 months, will be given in addition to the BI. The CBT builds on the message contained in the BI, and is theoretically based on Chalder's CBT model for CFS patients [17], and on the Cognitive Activation Theory of Stress (CATS) [5]. This CBT model assumes that cognitive, emotional, physiological, and behavioral responses are linked, and that changes in one of these areas will result in changes in others. During the treatment, patients are helped to change their interpretation of the pain and associated fear, symptom focusing, and avoidance. Participants are encouraged to see complaints as temporary and reversible, and not as signs of harm, or as evidence of a permanent condition or as a fixed disease pathology. The aim of the intervention is to help patients change cognitive- and behavioral factors assumed to be partly responsible for the maintenance of symptoms and disability. Return to work is an outlined goal of the treatment.

A treatment manual has been written by Reme & Chalder [63], and developed in close collaboration with experienced specialists in family medicine, psychiatry, and physical medicine and rehabilitation, all educated in cognitive behavioral therapy (Egil Fors, Tone Tangen, Peer Staff and Jens Ivar Brox). LBP patients seem to be sensitive to conflicting messages. Even small changes in the information given, may bring back the fear and avoidance behavior [64]. This requires both a detailed manual, and consistent training and supervision of the clinicians. The CBT-clinicians in this study are all health care personnel with varied backgrounds; six MD's with a specialty in physical medicine and rehabilitation, two psychologists, one psychiatric nurse, and one physiotherapist. All had to undergo a general CBT-course (with a minimum duration of 10 days and 15 hours of group supervision), or be familiar with CBT from previous education and training. In addition, all CBT-clinicians received three days of training in this LBP-tailored CBT, followed by at least one individual session with a patient not included in the study. The session is recorded on videotape, and rated by the supervisors and the first author of the treatment manual (SER). The session will be rated according to protocol adherence, and satisfying quality of the CBT. All clinicians have to be approved before treating patients included in the trial. All CBT sessions in the trial will be audio taped. Tapes will be used for supervision as well as rating for research purposes. Supervision is offered approximately every 4^th ^month, but the clinicians are offered phone and email contact with supervisors when needed. A random selection of the tapes are continuously evaluated by independent raters (SER and graduate students in psychology), with a percentage evaluated twice for inter rater reliability, using a modified Norwegian version of the Cognitive Therapy Adherence and Competence Scale [65].

#### 3. Nutritional supplements (seal oil and soy oil)

Findings from patients with inflammatory bowel disease (IBD) suggest that seal oil may reduce musculoskeletal pain. IBD patients report high levels of comorbidity, including musculoskeletal pain [66]. A substantial amelioration of joint pain after short-term administration of seal oil, has been reported in one pilot study [35], and one controlled study [36]. In Bjørkkjær et al. [67] prostaglandin E_2 _(PGE_2_) levels were also reduced. Given these findings, testing a possible effect of seal oil in LBP patients seems a reasonable next step, and an ethically acceptable experiment. In addition, with the current interest in alternative medicine in the Norwegian population, seal oil seems to have high face validity as a potential effective supplement for muscle pain.

Patients randomized to nutritional supplements, will receive commercially available seal oil for the same duration as the CBT treatment, in a double-blind randomized, controlled design. Oils will be administered as 20 capsules daily, providing 638 mg EPA, 399 mg DPA, and 798 mg DHA per day for those receiving seal oil, and 5.31 g linoleic acid (18:2n-6) per day for those receiving soy oil. Before meals, 10 capsules should be taken in the morning and 10 capsules in the evening, with fluid. Capsules will be stored at room temperature in blinded light-protected boxes during the study. Capsules are chosen because they are easy to administrate, have no taste, less regurgitation problems, and are easier to blind, compared to using the oils in liquid form. Testing of the oils before the intervention showed that vitamin A, i.e. sum retinol (13-, 11-, 9-cis) and all trans retinol, i.e. A_1 _and 3,4 didehydro-all-trans retinol (A_2_) in both oils were below 0.28 mg/kg. Vitamin D_3 _content in daily soy oil and seal oil dosages were 1 μg and 0 μg respectively. The antioxidant D-alpha-tocopherol was added in the case of seal oil, giving a vitamin E (alpha-tocopherol) content in seal oil and soy oil of 85.4 and 16.6 g/100 g respectively. The seal oil contains 56.6 g/100 g monounsaturated fatty acids, which are less prone to lipid peroxidation than polyunsaturated fatty acids (PUFAs), and the oil has no known major adverse effects [35,36]. Soy oil is common in the diet in the western world, and is considered a placebo in this trial. Cholesterol levels in seal oil and soy oil were 36.8 and 2.9 g/100 g respectively. The seal oil is a refined oil from the blubber of adult harp seals (*Phagophilus groenlandicus*). The soy oil is a refined oil from soybeans, which are GMO-free. Both oils were approved according to current legislations on contaminants and relevant quality standards

### Drop out from randomized treatment

Participants who drop out of treatment will be asked if they are willing to continue filling out questionnaires. Those who wish to continue contributing data to the study, but who are not able to or decline to come to the clinic, will receive the questionnaires by mail, together with pre-paid return envelopes.

### Assessment and Procedures

All participants will fill out questionnaires at the clinic immediately before BI. Follow up is conducted at 3, 6, and 12 months after the first session of BI. Data on sick leave will be obtained from the Norwegian National Insurance Administration (see outcome), and will also be collected 24 months after randomization (Figure 1).

### Measures

#### Primary outcome measures

The main outcome in the study is sick leave. The data on sick leave/insurance status will be based on register data from the Norwegian National Insurance Administration.

#### Secondary outcome measures

##### Questionnaires

Standard forms contain items for demographics, medication, previous treatments, pain intensity, and life style variables. Below follows a short description of the most important questionnaires, all validated and in Norwegian:

1. Subjective Health Complaints (SHC): The SHC-inventory records complaints without asking for attributions or medical diagnosis [68]. The selection of items is not based on any specific theory, but covers the most frequent health complaints and reasons for being seen by a general practitioner [69]. The inventory has 5 subscales; musculoskeletal, pseudoneurological, gastrointestinal, flu, and allergy complaints, and covers the period of the previous 30 days. The reliability and validity are good [68].

2. Illness Perception Questionnaire Revised (IPQ-R): This scale has five subscales providing information on the five components underlying the cognitive representation of the illness. These are: identity (the symptoms that the patient associates with the illness), cause (personal ideas about etiology), timeline (the perceived duration of the illness), consequences (expected effects and outcome) and cure-control (how one controls or recovers from the illness). The internal consistency, validity, and test-retest reliability of the separate scales have been found to be good [70].

3. Oswestry Disability Index [71] and Norwegian Function Assessment Scale [72] both assesses functional limitation. Reliability and validity of the Oswestry Disability Index have been found to be good [73]. The Norwegian Function Assessment Scale has 7 sub domains, and reliability and validity have been tested and found good [74].

4. Hospital Anxiety and Depression Scale (HAD) and Hopkins Symptom Checklist (HSCL) both assesses mood. The HAD scale has been found to be a reliable instrument for detecting states of depression and anxiety, and the anxiety and depressive subscales are also valid measures of severity of the emotional disorder [75]. The scale avoids overlap with somatic symptoms of physical illness. The HSCL-25 scale consists of 25 questions about the presence and intensity of the most common symptoms of anxiety and depression [76].

5. Chalder Fatigue Scale: This is an 11-item scale measuring physical and mental fatigue. The sum score can range from 0 to 33; a bimodal score ranging from 0-11 can also be obtained. The fatigue scale has been used extensively in research and has good psychometric proprieties [77]. It has also been validated in Norwegian [78].

6. Fear Avoidance Beliefs Questionnaire (FABQ): This is a scale that measures self-reported fear avoidance thoughts and beliefs in LBP patients [79]. It has 2 subscales; fear avoidance for physical activity and fear avoidance for work. Reliability and validity was tested in a Norwegian population of LBP patients and found to be acceptable [80].

7. Pain Stages of Change Questionnaire (PSOCQ): This is a scale measuring motivation to change. It is a multidimensional instrument designed to measure readiness to change in individuals with chronic pain [81]. Five stable subgroups have been identified and named: Pre-contemplation, contemplation, non-contemplative action, participation, and the ambivalent subgroup [82].

8. A mini-version self-administrated food frequency questionnaire (FFQ) has been developed, with focus on habitual intake (i.e. last year) of fish and other seafood for dinner, as sandwich spread, in salads or as snack. The questions relate to type of seafood, frequency of intake and in some cases portion size. It also includes questions about the use of long chain omega-3 PUFA supplements (fish oil, seal oil etc.).

##### Psychiatric Interview

The Mini-International Neuropsychiatric Interview (MINI) was applied as the structured diagnostic interview [58] for DSM-IV [83] and ICD-10 [84] assessing psychiatric disorders. It is a short structured diagnostic interview, developed jointly by psychiatrists and clinicians in the United States and Europe. It is based on "yes" and "no" answers and covers 23 Axis 1 disorders. In the multiaxial system of DSM-IV, Axis I disorders include all major mental disorders as well as developmental and learning disorders. The MINI interview usually takes 15-20 minutes, and has high reliability and validity [58]. We used the Norwegian version of MINI Plus [85].

### Blood samples

For a selected number of participants, fasting venous blood samples are drawn and stored in -80°C freezer for later analysis, or in a -20°C freezer for 4 weeks, before subsequent rapid analysis. The samples will be taken before and after the interventions, using validated methods at the NIFES [86] with modifications. Fatty acid composition will be measured in unwashed erythrocytes, to get information on compliance, and possible corroboration to any treatment effect. Extra vials of unwashed erythrocytes and plasma at inclusion and after 3 months will be stored in a bio bank for a possible future balanced risk assessment (nutrients and contaminants in same sample) of seafood intake.

### Saliva samples

Previous studies have shown that cortisol measured through saliva using salivette, is a reliable and valid measure of unbound and free cortisol level in plasma [87]. Saliva cortisol will be measured in order to assess the cortisol day curve (stress profile). Furthermore, the aim is to investigate if the different treatments affect the cortisol profile. The participants collect saliva on two consecutive days between Tuesday and Friday at baseline and 3 months later. Four saliva samples will be collected each day; immediately after awakening, 30 minutes later, and at 3 pm and 10 pm. Participants will be asked to avoid food, drinks and nicotine 30 minutes before collecting saliva, and keep a written record of the exact time of awakening and saliva collecting. The participants keep the saliva samples in their freezer at home, before bringing them to the laboratory for analyses.

### Analyses

#### Sample size calculations

The sample size calculations were based on data from Hagen et al. [30] and the transition probabilities calculated for the intervention group in that study. The sample size was based on the transition probabilities at 9 months, where approximately 40% of the individuals had returned to work. Hagen et al. [59] found that the effects were largest for the earlier period after the intervention. This is therefore an argument to use 40% off sick leave at 9 months as the most relevant measure for effect in this study. This was compared to a hypothesized 60% return to work (RR 1.5) as an adequate effect size. The sample size calculations were based on standard formulas for calculating sample size, for studies comparing binomial proportions using the statistical package "R" (Library Hmisc). This may increase the power of the estimates in the analyses, since the simpler method used for the calculation does not include in this issue. All calculations were based on a power of 80% and a significance level of 5%.

Separate sample size calculations for males and females were computed. The total number of individuals should be approximately N = 4*n, where n is the calculated sample size and 4 is the number of arms in the study. The total number of males should be N = 4*65 = 260 and the total number of females N = 4*81 = 324. If we control for gender in the analysis instead of performing stratified analyses, we do not need to calculate separate sample sizes for men and women. The total number of participants should be 97 in each arm (N = 388). These numbers require a multi-centre design.

### Analysis strategy

#### Primary analyses of efficacy

Sick leave/insurance status will be calculated as risks of shifting between different states of sick leave, disability pension, and recovered. The outcome from the participating clinics will be combined if no gross center effect is found. The analyses will follow the "intention to treat" principle. Per protocol analysis will also be performed. The proportions of participants with a positive primary outcome will also be compared across the treatment groups. If there are significant differences, we will estimate the number of participants that need to be treated in order for one additional participant to benefit (NNT), compared to the least effective therapy. A Cox-proportional hazards model may model the transitions between the different states. Regression models for the transition probabilities will also be performed. Explanatory variables (treatment) and confounders (e.g. age, gender) will be included as in any regression model. Possible changes in diagnosis and other characteristics will be built in. Partial recovery represented by partial sick leave will be accounted for.

#### Secondary analyses of efficacy

Any significant effects of any of the interventions will be estimated by a standard cost benefit formula [88-90], as used by Hagen et al [59]. Benefits will be measured in terms of increases in the net present social value of production, when intervention causes a reduction in the number of days on sick leave. This is calculated as the product of the treatment effect, i.e. the reduction in sick leave days, and the productivity gains for the society when a person works instead of receiving benefits. For each person the social value of production is based on mean earnings at inclusion in the treatment group. Cost of treatment also includes follow-up at the clinics. Potential differences in treatment costs between the groups, which are related to treatments received independent of the study, will be accounted for by follow-up questionnaires from the participants.

We will also evaluate health complaints/pain and disability as recorded by the questionnaires at 3, 6 and 12 months. Appropriate regression models, based on the distribution for the different outcomes, will be performed, including variables found to be statistically significant predictions.

#### Predictions and process of treatment

Associations between post-treatment outcomes and predictor variables will be examined, using multiple linear and logistic regression modeling techniques.

#### Independent overseers

The independent Scientific Advisory Board, and the Trial Steering Committee, provides supervision for the trial and safeguards its integrity, including data monitoring and possible ethical challenges. In addition, The Regional Ethical Committee and the Norwegian Social Science Data Services have approved the study.

#### Confidentiality

All data collected is regarded as confidential and securely stored in paper-formats, and on memory sticks. The memory sticks and all data containing personal information are stored in a fireproof safe.

#### Therapists' compliance with treatment manuals

Following and checking audio recordings will monitor therapist compliance with treatment manuals. Any significant deviations from the manual will be noted and feedback given to the therapist. Therapists are allowed to treat trial participants once they have been approved as competent. Every fifth BI and CBT session will be randomly chosen and evaluated independently by an assessor to assess adherence to the manual.

## Discussion

To our knowledge, the CINS trial will be the largest randomized trial where brief intervention and cognitive behavioral therapy are compared to nutritional interventions for LBP. It will provide important information about effectiveness of the targeted interventions, possible adverse effects, cost-effectiveness, prevalence, predictors, and mechanisms of change. The results will provide significant information to patients, health care providers and commissioners about which treatment works for whom.

## Competing interests

The authors declare that they have no competing interests.

## Authors' contributions

HRE and TC designed the study, and are the principal investigators. HRE, SER, and THT wrote the manuscript on behalf of the CINS-project. TC revised and commented on the manuscript. SER is the coordinator of the study and drafted the first version of the manuscript. THT participated in the design and coordination of the study. TB participated in the design of the study, contributed with his expertise on the nutritional supplements, and revised and commented on the manuscript. AI participated in the design of the study, in the development of BI, and commented on the manuscript. JIB participated in the design of the study, and contributed to the development and evaluation of treatment protocols, and commented on the first and final manuscript. EF participated in the training of the CBT therapists, and commented on the manuscript. EMH participated in the design of the study, in the development of BI, and commented on the manuscript. All authors read and approved the final manuscript.

## Contributors

Stein Atle Lie did the sample size calculations and contributed in developing the strategy for data analysis. Tone Tangen participated in the training and supervision of the CBT-therapists. Astrid Grasdal and Jan Erik Askildsen planned the strategy for the cost/benefit analyses. Anette Harris and Holger Ursin contributed with the strategy for sampling and analysis of cortisol.

## Funding

The Norwegian Research Council (NFR) contributed with the main funding for the study. Helse Vest funded one research fellow working with the project, and the University of Bergen funded one research fellow and one postdoctoral research fellow working with the project. Their home institutions financed the researchers and clinicians participating in the project. G.C. Rieberfondene donated funds for the daily run of the study and Mills DA donated the soy oil.

## Pre-publication history

The pre-publication history for this paper can be accessed here:

http://www.biomedcentral.com/1471-2474/12/152/prepub
